# Balanced steady-state free precession MRCP is a robust alternative to respiration-navigated 3D turbo-spin-echo MRCP

**DOI:** 10.1186/s12880-020-00532-w

**Published:** 2021-01-11

**Authors:** Felix Christian Hasse, Buket Selmi, Hamed Albusaidi, Theresa Mokry, Philipp Mayer, Christian Rupp, Hans-Ulrich Kauczor, Tim Frederik Weber

**Affiliations:** 1 Diagnostic and Interventional Radiology, Heidelberg University Hospital, Im Neuenheimer Feld 110, 69120 Heidelberg, Germany; 2 Department of Gastroenterology, Heidelberg University Hospital, Im Neuenheimer Feld 410, 69120 Heidelberg, Germany

**Keywords:** Cholangiopancreatography, Magnetic resonance, Biliary tract, Pancreas

## Abstract

**Background:**

Despite synchronization to respiration, respiration-navigated (RN) 3D turbo-spin-echo MRCP is limited by susceptibility to motion artifacts. The aim of this study was to assess the quality of pancreaticobiliary duct visualization of a non-RN MRCP alternative based on balanced steady-state free precession imaging (BSSFP) with overlapping slices compared with RN-MRCP.

**Methods:**

This is a retrospective study on 50 patients without pancreaticobiliary duct disease receiving MRCP at 1.5 T. We performed an intraindividual comparison of coronal RN-MRCP with combined coronal and transverse BSSFP-MRCP. Image quality was scored by 3 readers for 6 pancreaticobiliary duct segments (3 pancreatic, 3 biliary) using a 6-point scale. A segment score of 3 or lower as assessed by at least 2 of 3 readers was defined as insufficient segment visualization. Nonparametric tests and interrater reliability testing were used for statistical analysis.

**Results:**

Overall duct visualization averaged over all readers was scored with 4.5 ± 1.1 for RN-MRCP (pancreatic, 4.1 ± 0.5; biliary, 5.0 ± 0.4) and 4.9 ± 0.9 for combined coronal and transverse BSSFP-MRCP (pancreatic, 4.6 ± 0.6; biliary, 5.1 ± 0.6), respectively (*p* < 0.001). The number of segments visualized insufficiently was 81/300 for RN-MRCP and 43/300 for BSSFP-MRCP (*p* < 0.001). Segments visualized insufficiently only in RN-MRCP had a mean score of 4.4 ± 0.8 in BSSFP-MRCP. Overall interrater agreement on superiority of BSSFP-MRCP segment scores over corresponding RN-MRCP was 0.70. Mean acquisition time was 98% longer for RN-MRCP (198.0 ± 98.7 s) than for combined coronal and transverse BSSFP-MRCP (100.2 ± 0.4 s).

**Conclusions:**

Non-RN BSSFP-MRCP with overlapping slices is a fast alternative to RN-MRCP, frequently providing sufficient duct visualization when RN-MRCP fails.

## Background

Magnetic resonance cholangiopancreatography (MRCP) is a non-invasive imaging technique to assess the biliary and pancreatic duct system. Early MRCP studies already employed balanced steady state free precession (BSSFP) sequences [1, 2]. In contemporary clinical practice, however, MRCP is commonly performed using 3D respiration-navigated T2 turbo spin echo sequences (RN-MRCP). The diagnostic performance of this latter imaging technique is considered equal to that of the gold standard in many cases, i.e. ERCP [3]. Synchronization of image acquisition to respiration yields better image quality and higher diagnostic value compared to breathhold acquisition in 3D T2 turbo spin echo sequences [4, 5]. Using RN-MRCP, a high spatial resolution with nearly isotropic voxel sizes can be achieved [6–9]. Despite synchronization to respiration, the relatively long acquisition time renders RN-MRCP susceptible to motion artifacts [10]. Insufficient image quality due to diaphragmatic shift is common in patients who are unable to breathe steadily [11].

2D BSSFP imaging is characterized by a short TR and high signal-to-noise ratio (SNR). Due to its short acquisition time, BSSFP-MRCP can be performed in shallow breathing. Moreover, it is feasible to acquire overlapping slices with little saturation effects [12, 13]. In combination with a bright rendering of fluids these features make it a potential alternative for MRCP.

The aim of this exploratory study was to assess the quality of pancreaticobiliary duct visualization of BSSFP-MRCP compared with RN-MRCP. For this we conducted a multi-reader assessment of pancreaticobiliary duct visibility in patients without evidence of pancreaticobiliary duct disease.

## Methods

### Study design

This is a retrospective single-center exploratory study that was approved by the institutional review board with a waiver of informed consent.

Inclusion criteria were patient age ≥ 18 years and availability of MRCP including both RN-MRCP and BSSFP-MRCP during the study period lasting from September 2017 to May 2018. Exclusion criterion was evidence of a pancreaticobiliary abnormality that per se precluded full assessment of the pancreaticobiliary duct system, such as history of pancreaticobiliary surgery, pancreaticobiliary duct stones, presence of solid pancreatic or biliary benign or malignant masses and cystic lesions of the pancreas ≥ 10 mm. MRI with MRCP was performed based on clinical indications at the discretion of the referring physician.

### Study population

The study population consisted of 50 consecutive patients (28 female, 22 male; mean age 52.02 ± 14.78, range 22–78 years). In these, the most common indications for MRCP were exclusion of abdominal masses (n = 15), exclusion of PSC (n = 12), unclear hepatopathy (n = 11), suspected inflammatory disease of the pancreas (n = 6), suspected Intraductal Papillary Mucinous Neoplasia (n = 4), and others (n = 2). Following our standard operating procedures, all patients were asked to come to their examination appointment on an empty stomach. No other preparation was required.

### Imaging

MRCP examinations were performed using a 1.5 T scanner (Magnetom AvantoFit, Siemens Healthineers). Patients fasted for a minimum of 6 h prior to the examination. In accordance with the standard clinical protocol, neither spasmolytic drugs nor negative oral contrast agents were administered.

The MRCP protocol included a state-of-the-art coronal RN-MRCP sequence with a slice thickness of 1.5 mm. Respiration navigation was achieved using prospective acquisition correction. The standard RN-MRCP was followed by two BSSFP-MRCP sequences, one in transverse orientation with a slice thickness of 6 mm and 3.6 mm overlap and one in coronal orientation with a slice thickness of 4 mm and 2.4 mm overlap. For BSSFP-MRCP, patients were instructed to hold their breath for a comfortable period of time followed by shallow breathing. The acquisition times of the sequences were recorded. For an overview of the sequence data see Table 1.

**Table 1:** MRCP sequence protocol data

	RN	BSSFP trans	BSSFP cor
Slice thickness (mm)	1.5	6.0	4.0
Interval (mm)	1.5	2.4	1.6
TR (ms)	2000	538	520
TE (ms)	686	2	1
Acceleration factor	3	2	2
Overlap	0%	60%	60%

RN, respiration-navigated MRCP; BSSFP trans, balanced steady state free precession MRCP in transverse orientation; BSSFP cor, balanced steady state free precession MRCP in coronal orientation

### Image assessment

Duct visualization was scored by 3 independent readers for 3 pancreatic and 3 biliary duct segments. Readers 1, 2 and 3 had 8 years, 5 years and 2 years of experience in abdominal radiology, respectively. Head, body, and tail were defined as the 3 pancreatic segments. The 3 biliary segments were the common bile duct (CBD) as well as the left hepatic duct and the right hepatic duct up to their first branching.

Visibility of these 6 segments was judged on a 6-point scale (duct visibility score): 1, not depicted; 2, uninterpretable; 3, identifiable < 50%; 4, identifiable > 50% but < 100%; 5, entirely depicted with blurring; 6, entirely depicted with excellent details. A 5-point scale was used for rating the quality of the MRCP images concerning the presence of motion artifacts (artifact score): 1, non-diagnostic image due to severe artifacts; 2, major artifacts causing significant problems in diagnosis; 3, moderate artifacts with some uncertainty in diagnosis; 4, minor artifacts without problems in diagnosis; 5, excellent image quality without any detectable artifacts. For the combination of transversal and coronal BSSFP-MRCP, the better score of the two sequences was used.

All readers underwent a training session of 10 patients not included in the study prior to the first reading session. The reads were performed in 2 sessions, one for coronal RN-MRCP images and one for both transverse and coronal BSSFP-MRCP sequences, with a 2-week interval to minimize recall bias. Readers were blinded to clinical information. During the reading sessions, the examinations were presented to the readers in a randomized manner. Readers were allowed to consult a routine T2-weighted sequence without fat saturation in transverse orientation for anatomical orientation when assessing RN-MRCP. No maximum intensity projections were used.

A duct visibility score of 3 or lower as assessed by at least 2 of 3 readers was defined to indicate insufficient segment visualization.

### Statistics

The Wilcoxon signed-rank nonparametric test was used for statistical analysis of differences between groups, *p* < 0.05 was considered to be statistically significant. Bonferroni correction of *p* values was employed to adjust for multiple comparisons. For the assessment of interrater reliability, the Fleiss Kappa test was performed. The individual segment scores of BSSFP-MRCP were reviewed for superiority over RN-MRCP. The binary values superiority and non-superiority of BSSFP-MRCP were then tested for reliability between the readers. κ values were interpreted in keeping with Landis and Koch [14] (0.21–0.40, fair agreement; 0.41–0.60, moderate agreement; 0.61–0.80, good agreement; 0.81–1.00, excellent agreement). Spearman correlation was determined for the non-metric data. The software used for statistical analysis was SPSS (version 25, IBM).

## Results

### Overall duct visualization

The overall duct visibility score averaged over all readers was 4.5 ± 1.1 (mean ± standard deviation) for RN-MRCP (pancreatic, 4.1 ± 0.5; biliary, 5.0 ± 0.4). For transverse and coronal BSSFP-MRCP, the overall duct visibility score was 4.6 ± 0.8 (pancreatic, 4.4 ± 0.7; biliary 4.8 ± 0.9) and 4.6 ± 0.8 (pancreatic, 4.3 ± 0.8; biliary 4.9 ± 0.8), respectively. The combination of transverse and coronal BSSFP sequences yielded a duct visibility score of 4.9 ± 0.9 (pancreatic, 4.6 ± 0.6; biliary, 5.1 ± 0.6). Combined transverse and coronal BSFFP-MRCP had a significantly higher score than RN-MRCP and single plane BSSFP-MRCP (*p* < 0.01). Single plane BSSFP-MRCP duct visibility scores were not significantly higher than RN-MRCP scores. The difference in pancreatic segment scores between RN-MRCP and combined transverse and coronal BSSFP-MRCP was statistically significant (*p* < 0.01), not so the difference between biliary segment scores. Individual overall duct visibility scores by reader were 4.3 ± 1.1, 4.2 ± 1.1, and 5.0 ± 1.0 for RN-MRCP and 4.8 ± 0.8, 4.3 ± 0.8, and 5.5 ± 0.7 for BSSFP-MRCP for Reader 1, 2 and 3, respectively. In 19% of all segment evaluations, the consultation of a second plane increased the overall score of BSSFP-MRCP. For an overview of pancreatic, biliary and overall reading scores see Table 2. Of the 6 defined segments, the highest scoring segment in both techniques was the CBD with 5.4 ± 1.0 in RN-MRCP and 5.6 ± 0.6 in BSSFP-MRCP. The lowest scoring segment in both techniques was the caudal pancreatic duct with 3.2 ± 1.5 in RN-MRCP and 4.1 ± 1.0 in BSSFP-MRCP. For segmental scores see Table 3.

**Table 2:** Overall, pancreatic, and biliary duct visualization scores

	Overall				Pancreatic				Biliary			
	RN	BSSFP trans	BSSFP cor	BSSFP comb	RN	BSSFP trans	BSSFP cor	BSSFP comb	RN	BSSFP trans	BSSFP cor	BSSFP comb
All Readers	4.5 ± 1.1	4.6 ± 0.8	4.6 ± 0.8	4.9 ± 0.9	4.1 ± 0.5	4.4 ± 0.7	4.3 ± 0.8	4.6 ± 0.6	5.0 ± 0.4	4.8 ± 0.9	4.9 ± 0.8	5.1 ± 0.6
Reader 1	4.3 ± 1.1	4.4 ± 0.9	4.6 ± 0.9	4.8 ± 0.8	3.9 ± 1.3	4.0 ± 1.1	4.2 ± 1.2	4.4 ± 1.2	4.7 ± 1.1	4.7 ± 1.0	4.9 ± 1.0	5.1 ± 0.8
Reader 2	4.2 ± 1.1	3.9 ± 0.8	3.9 ± 0.9	4.3 ± 0.8	3.7 ± 1.2	3.9 ± 0.9	3.6 ± 0.9	4.1 ± 0.9	4.7 ± 1.1	3.9 ± 1.0	4.2 ± 1.1	4.5 ± 0.9
Reader 3	5.0 ± 1.0	5.5 ± 0.7	5.5 ± 0.7	5.5 ± 0.7	4.6 ± 1.3	5.2 ± 1.0	5.2 ± 1.0	5.3 ± 1.0	5.5 ± 1.0	5.7 ± 0.6	5.8 ± 0.6	5.8 ± 0.6

BSSFP trans refers to transverse BSSFP imaging scores, BSSFP cor to coronal BSSFP imaging scores and BSSFP comb to the combined score of transverse and coronal. Values are given as mean ± standard deviation

**Table 3:** Segmental duct visualization scores

Segment	1		2		3		4		5		6	
	RN	BSFFP	RN	BSFFP	RN	BSFFP	RN	BSFFP	RN	BSFFP	RN	BSFFP
All Readers	4.8 ± 1.3	5.1 ± 0.9	4.2 ± 1.3	4.6 ± 1.1	3.2 ± 1.5	4.1 ± 1.0	5.4 ± 1.0	5.6 ± 0.6	5.1 ± 1.1	5.0 ± 0.8	4.5 ± 1.5	4.8 ± 0.8

Segment 1, pancreatic duct (PD) head; segment 2, PD body; segment 3, PD tail; segment 4, CBD; segment 5, left hepatic duct; segment 6 right hepatic duct. BSSFP refers to the combined score of transverse and coronal BSSFP-MRCP. Values are given as mean ± standard deviation

**Fig. 1:**
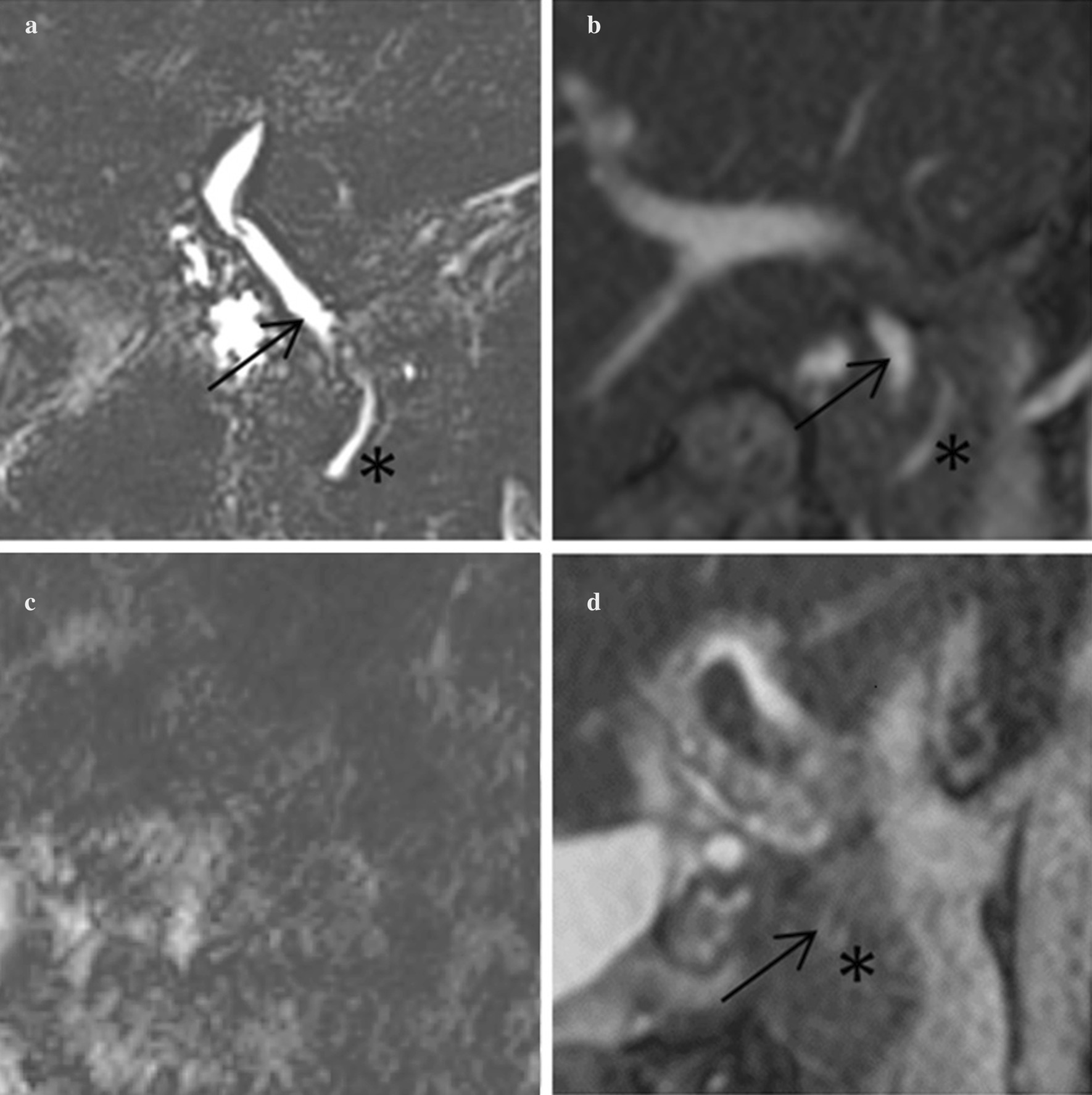
Examples of duct visualization. **a** Coronal RN-MRCP in patient 1; **b** coronal BSSFP-MRCP in patient 1; **c** coronal RN-MRCP in patient 2; coronal BSSFP-MRCP in patient 2; arrows point at the CBD, asterisks mark the pancreatic duct, if identifiable. Patient 1 achieved excellent visualization scores in both imaging techniques with little to no artifacts. In patient 2 duct visualization failed in RN-MRCP due to severe artifacts, however the duct system could be visualized with BSSFP-MRCP

**Fig. 2:**
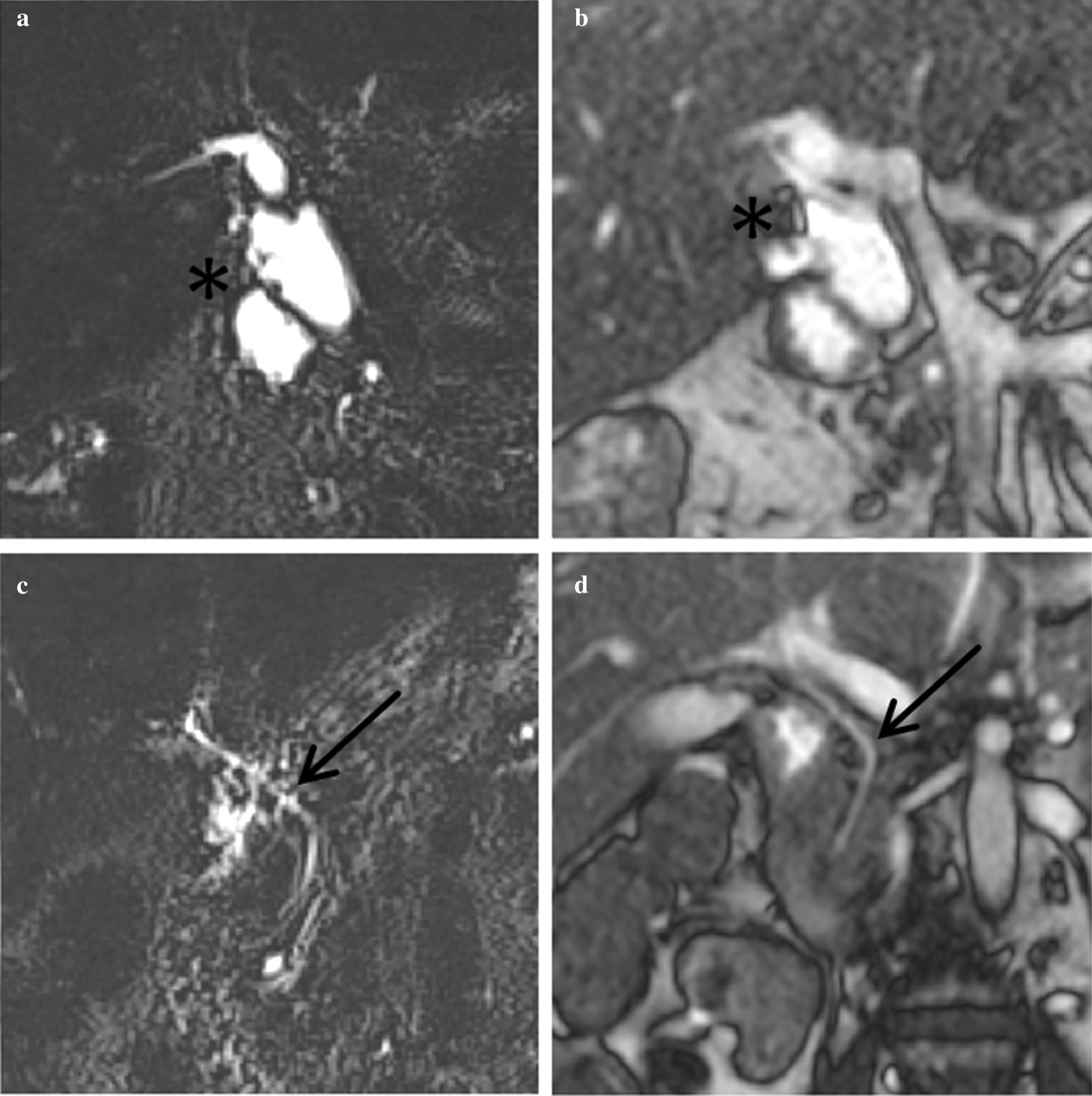
Metal and fluid artifacts. **a** Coronal RN-MRCP in patient 3; **b** coronal BSSFP-MRCP in patient 3; **c** coronal RN-MRCP in patient 4; **d** coronal BSSFP-MRCP in patient 4; asterisks mark the location of a metal clip, arrows point at the CBD. The metal clip can only be delimited clearly in BSSFP-MRCP. Fluid signal from the pyloric antrum merges with the CBD signal only in RN-MRCP in patient 4 in absence of relevant motion artifacts. In BSSFP-MRCP image quality in patient 4 is not compromised by gastric fluid

Motion artifacts were significantly less prevalent in BSSFP-MRCP with an artifact score of 4.0 ± 0.4 for both transverse and coronal BSSFP-MRCP versus 3.5 ± 0.9 for RN-MRCP (*p* < 0.001). For RN-MRCP, there was a strong positive correlation between low visualization scores and motion artifacts as the two scores yielded a Spearman coefficient of 0.67 (*p* < 0.001). Neither for transverse nor for coronal BSSFP-MRCP a statistically significant correlation between low visualization scores and motion artifacts was observed. There was no significant correlation between patient age or sex and prevalence of artifacts. Furthermore, there was a moderate, statistically significant correlation between artifact scores of RN-MRCP and BSSFP-MRCP with a Spearman coefficient of 0.36 (*p* = 0.01). For exemplary images of RN-MRCP with and without motion artifacts and BSSFP-MRCP see Fig. 1. A consensus read showed that causes other than motion artifacts influenced the visualization of the pancreaticobiliary duct system only in 4 of 50 cases. In two cases, this was due to an unfavorably positioned clip after cholecystectomy and in two cases due to excessive filling of the stomach/duodenum. In the former cases, a clearer delimitation of the metal artifacts by the clip material in the BSSFP-MRCP was observed, whereas in the RN-MRCP the metal artifacts could not be distinguished from the environment (Fig. 2). In one case, fluid overlays made it difficult to delimit sections of the CBD in RN-MRCP, while the visualization in BSSFP-MRCP was not affected (Fig. 2). There were no other relevant sources of artifacts like excessive gas or ascites in the study population.

### Insufficiently visualized segments

**Fig. 3:**
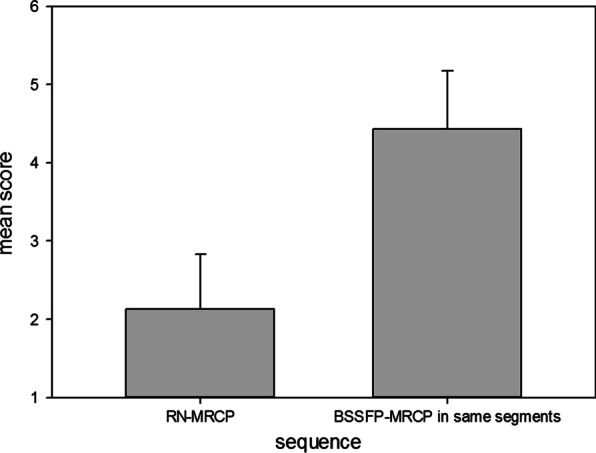
Visualization score comparison. Score of 49 insufficiently visualized segments in RN-MRCP (score < 3) and the corresponding score of the same segments in combined transverse and coronal BSSFP

According to the definition given above, the number of segments visualized insufficiently in only one of the imaging techniques was 49/300 for RN-MRCP and 11/300 for BSSFP-MRCP (*p* < 0.001). In addition, 32 segments were visualized insufficiently in both sequence techniques. Segments visualized insufficiently in RN-MRCP had a mean score of 4.4 ± 0.8 in BSSFP-MRCP (see Fig. 3). The segment most frequently visualized insufficiently was the pancreatic tail for both RN-MRCP (29/50) and BSSFP-MRCP (16/50). An average patient score (all segment scores in one patient averaged) of 3 or lower was seen by at least 2 readers in 7/50 patients in RN-MRCP and only in 1/50 patients in BSSFP-MRCP.

There were 11 segments scored as not depicted (duct visualization score = 1) in RN-MRCP and none in BSSFP-MRCP. The pancreatic tail and the right hepatic duct were the only segments in which absence of depiction occurred. 10 out of these 11 segments were sufficiently visualized in BSSFP-MRCP. 64% of patients had at least one insufficiently visualized duct segment in RN-MRCP compared to 44% for BSSFP-MRCP.

### Interreader agreement

Fleiss Kappa for superiority of BSSFP-MRCP segment scores over corresponding RN-MRCP was 0.70 (*p* < 0.001). There was no significant difference in agreement between the first 25 and second 25 patients (*p* > 0.05). Thus, no increase in agreement could be observed over the course of the study.

### Acquisition time

Mean acquisition time was 98% longer for RN-MRCP with 198.0 ± 98.7 s than for combined coronal and transverse BSSFP-MRCP with 100.2 ± 0.4 s (*p* < 0.001).

## Discussion

The present study is the first multi-reader quantitative comparison of pancreaticobiliary duct visualization in RN- and BSSFP-MRCP. In our study, RN-MRCP was significantly outperformed by the almost twice as fast combination of transverse and coronal BSSFP-MRCP concerning mere pancreaticobiliary duct visualization. The combination of transverse and coronal BSSFP-MRCP was superior to single-plane BSSFP-MRCP. The key difference between RN-MRCP and BSSFP-MRCP was the visualization of the pancreatic duct whereas there was no statistically significant difference in visualization of the biliary duct system. Issues in RN-MRCP were a relatively high rate of segment non-depiction and susceptibility to motion artifacts. The quantitative results regarding susceptibility to motion artifacts are in keeping with the impression of Glockner et al. [13]. However, the overall result of the present study differs from previous ones in that the latest BSSFP-MRCP sequences can perform better than conventional RN-MRCP in terms of duct visualization, therefore coming closer to being an alternative.

There were no non-depictions of ducts in BSSFP-MRCP making this method more robust than RN-MRCP. Insufficient duct visualization can be a reason to repeat an MRCP examination, thus consuming valuable resources. Sufficient pancreaticobiliary duct visualization is the basis for differentiating between normal anatomy, norm variants and pathological findings. Especially in patients with non-depiction of ducts in RN-MRCP, BSSFP-MRCP can be used to generate sufficient images with little prolongation of the acquisition time. However, it must be acknowledged that in duct segments of patients with severe motion artifacts both sequences could not produce sufficient duct visualization, especially in the fine duct of the pancreatic tail. Sources of artifacts other than motion had no relevant impact on the study population. Artifacts from intraintestinal fluid and gas were reduced by examining patients on an empty stomach. Low prevalence of other sources of artifacts may also have been due to a relatively healthy study population by exclusion of a wide range of pathologies. Glockner et al. evaluated metal and gas artifacts as more interfering in BSSFP-MRCP than in RN-MRCP [12]. This statement cannot be assessed due to the small number of these artifacts in the present study population. Nevertheless, it should be noted that a metal artifact is unlikely to be mistaken as a filling defect in a duct in BSSFP-MRCP due to the clear demarcability of the metal.

In this study, we compared coronal RN-MRCP to transverse and coronal BSSFP-MRCP. Both transverse and coronal BSSFP sequences had to be consulted for a significantly better imaging score. While the two BSSFP sequences still had a significantly shorter acquisition time than RN-MRCP it could be argued that the reading time for two sequences is longer than for one, especially since there is more anatomical information in BSSFP sequences. For example, it can be challenging to follow bile ducts in BSSFP sequences along the hyperintense portal veins. At the same time, BSSFP is valuable in a preoperative setting because it offers information on biliary ducts and the vascular system in one sequence. A further benefit of the visualization of blood vessels in BSSFP sequences could be secondary vascular diagnose [15].

Several approaches have been developed to reduce banding artifacts, which ultimately made the application of BSSFP in MRCP expedient [15–22]. It has been established that BSSFP imaging is more resistant to flow artifacts while being more susceptible to chemical shifts, i.e. air or metal artifacts, in comparison to turbo spin echo imaging [12]. Overall, fewer artifacts occurred in BSSFP-MRCP than in RN-MRCP in the present study, as quantified by the artifact score.

The present study was focused on the visualization of the main duct segments of the pancreaticobiliary system. No deductions about the diagnostic value of the MRCP techniques with regard to structural diseases of the ducts or masses can be made since only patients without pathology of the pancreaticobiliary duct system were included. Although in exploratory studies numerous diagnoses could be made equally reliably using BSSFP-MRCP [13], the higher spatial resolution of RN-MRCP could play a pivotal role in identifying some pathologies, e. g. minor irregularities in PSC. Therefore, BSSFP-MRCP may not show the same superiority in diagnosing structural pancreaticobiliary duct disease as in duct visualization. Nevertheless, BSSFP-MRCP has been proposed as an alternative to conventional RN-MRCP in PSC diagnosis in patients with motion artifacts [12]. Until extensive studies have been conducted on the performance of BSSFP in different pathologies the authors will not generally recommend BSSFP-MRCP as an alternative to RN-MRCP. Both sequence techniques performed well in the pancreatic head and CBD, where pathologies like stones and anatomic variations like pancreas divisum can often be found.

Breathhold Compressed Sensing MRCP (CS-MRCP) and breathhold GRASE-MRCP have been proposed as even faster alternatives to RN-MRCP with good visualization [23, 24]. Acquisition times for these sequences can be well below 30 s in breath hold technique [23, 25, 26] and thus more than three times faster than the average BSSFP-MRCP sequence in the present study. Still, a long breath hold may not be achievable for many of the patients with poor results in RN-MRCP. Non-breathhold CS-MRCP is more time consuming than BSSFP-MRCP and with 132–228 s within the range of conventional RN-MRCP [23, 26–28]. Another issue is the conceptualization of both CS-MRCP and GRASE-MRCP for 3 T MRI. In the case of the former, this is now being contested with varying results for image quality of CS-MRCP at 1.5 T [29, 30]. Availability of CS-MRCP is further limited by its high processing power requirements. The issue of smaller duct visualization has been raised for CS-MRCP [31, 32] and GRASE-MRCP [25]. Different MRCP techniques are known to have different benefits in diagnosing pancreaticobiliary duct disease [33]. Therefore, more than one technique may be employed for optimal diagnostics.

Only the original slices of the two MRCP techniques were used for the reads. Therefore we did not employ negative oral contrast agents often used in thick slab MRCP to eliminate signal from overlapping fluid-containing bowel [34] or to improve the informative value of maximum intensity projections (MIP) reconstructed from 3D-MRCP. In functional diagnostics, MRCP can be supplemented with intravenous administration of secretin to stimulate pancreatic excretion [35]. However, there is no general recommendation for the use of secretin. At our institution secretin is only administered when functional information on pancreatic juice secretion is needed, i. e. pancreatic insufficiency and papilla stenosis and thus was not included in the protocol of the present study.

Limitations of this study are the small sample size and its single center approach. Only coronal slices of the RN-MRCP were used for the reads. Intrarater agreement was not determined. As discussed above, the diagnostic performance of both MRCP techniques was not evaluated. Since the underlying data of all reconstructions would be the same, no relevant impact on visualization scores was to be expected of consulting multiple reconstructions. Reader 3 scored segment visualization generally higher than Reader 1 and 2. The more experienced Readers 1 and 2 appeared to be more critical of image quality, being more restrictive with the distribution of the highest rating on the scale. This systematic deviation could most likely have been reduced by more extensive training sessions. To compensate for this, Fleiss Kappa testing was performed for superiority of BSSFP-MRCP over RN-MRCP and not for the absolute scores. No higher agreement was observed as scoring experience increased.

## Conclusions

In conclusion, combined transverse and coronal BSSFP-MRCP provided sufficient pancreaticobiliary duct visualization more reliably and in a shorter acquisition time than RN-MRCP. BSSFP-MRCP also yielded satisfactory images in many cases of patients with poor scores in RN-MRCP. Non-depiction of pancreaticobiliary duct segments is rare in BSSFP-MRCP. BSSFP-MRCP is considered a helpful accessory to RN-MRCP concerning the assessment of the pancreaticobiliary duct system.

## Data Availability

The datasets used and/or analysed during the current study are available from the corresponding author on reasonable request.

## Abbreviations

BSSFP: Balanced steady state free precession
CBD: Common bile duct
CS: Compressed sensing
GRASE: Gradient and spin echo
MRCP: Magnetic resonance cholangiopancreatography
PSC: Primary sclerosing cholangitis
RN: Respiration-navigated
T: Tesla
TR: Repetition time
